# Researching Mitigation of Alcohol Binge Drinking in Polydrug Abuse: KCNK13 and RASGRF2 Gene(s) Risk Polymorphisms Coupled with Genetic Addiction Risk Severity (GARS) Guiding Precision Pro-Dopamine Regulation

**DOI:** 10.3390/jpm12061009

**Published:** 2022-06-20

**Authors:** Kenneth Blum, Mark S. Brodie, Subhash C. Pandey, Jean Lud Cadet, Ashim Gupta, Igor Elman, Panayotis K. Thanos, Marjorie C. Gondre-Lewis, David Baron, Shan Kazmi, Abdalla Bowirrat, Marcelo Febo, Rajendra D. Badgaiyan, Eric R. Braverman, Catherine A. Dennen, Mark S. Gold

**Affiliations:** 1 The Kenneth Blum Behavioral & Neurogenetic Institute, Austin, TX 78701, USA; pathmedical@gmail.com (E.R.B.); catherine.a.dennen@gmail.com (C.A.D.); 2 Division of Addiction Research & Education, Center for Psychiatry, Medicine & Primary Care (Office of Provost), Western University Health Sciences, Pomona, CA 91766, USA; dbaron@westernu.edu (D.B.); febo@ufl.edu (M.F.); 3 Institute of Psychology, ELTE Eötvös Loránd University, Egyetem tér 1-3, 1053 Budapest, Hungary; 4 Department of Psychiatry, School of Medicine, University of Vermont, Burlington, VT 05405, USA; 5 Department of Psychiatry, Wright State University Boonshoft School of Medicine and Dayton VA Medical Centre, Dayton, OH 45324, USA; 6 Center for Alcohol Research in Epigenetics, Departments of Physiology and Biophysics, and Psychiatry, University of Illinois at Chicago, Chicago, IL 60612, USA; mbrodie@uic.edu (M.S.B.); scpandey@uic.edu (S.C.P.); 7 Molecular Neuropsychiatry Research Branch, National Institute on Drug Abuse, National Institutes of Health, Bethesda, MD 20892, USA; jcadet@intra.nida.nih.gov; 8 Future Biologics, Lawrenceville, GA 30043, USA; ashim6786@gmail.com; 9 Center for Pain and the Brain (P.A.I.N Group), Department of Anesthesiology, Critical Care & Pain Medicine, Boston Children’s Hospital, Boston, MA 02115, USA; dr.igorelman@gmail.com; 10 Behavioral Neuropharmacology and Neuroimaging Laboratory on Addictions, Clinical Research Institute on Addictions, Department of Pharmacology and Toxicology, Jacobs School of Medicine and Biosciences, State University of New York at Buffalo, Buffalo, NY 14203, USA; thanos@buffalo.edu; 11 Department of Psychology, State University of New York at Buffalo, Buffalo, NY 14203, USA; 12 Neuropsychopharmacology Laboratory, Department of Anatomy, Howard University College of Medicine, Washington, DC 20059, USA; mgondre-lewis@howard.edu; 13 College of Osteopathic Medicine of the Pacific, Western University of Health Sciences, Pomona, CA 91766, USA; shan.kazmi@western.edu; 14 Department of Molecular Biology and Adelson School of Medicine, Ariel University, Ariel 40700, Israel; bowirrat@gmail.com; 15 Department of Psychiatry, South Texas Veteran Health Care System, Audie L. Murphy Memorial VA Hospital, Long School of Medicine, University of Texas Medical Center, San Antonio, TX 78229, USA; badgaiyan@gmail.com; 16 Department of Psychiatry, Washington University School of Medicine, St. Louis, MO 63110, USA; drmarkgold@gmail.com

**Keywords:** risk polymorphisms, KCNK13, RASGRF2, genetic addiction risk severity (GARS), pro-dopamine regulation, binge alcohol drinking, K220

## Abstract

Excessive alcohol intake, e.g., binge drinking, is a serious and mounting public health problem in the United States and throughout the world. Hence the need for novel insights into the underlying neurobiology that may help improve prevention and therapeutic strategies. Therefore, our group employed a darkness-induced alcohol intake protocol to define the reward deficiency domains of alcohol and other substance use disorders in terms of reward pathways’ reduced dopamine signaling and its restoration via specifically-designed therapeutic compounds. It has been determined that KCNK13 and RASGRF2 genes, respectively, code for potassium two pore domain channel subfamily K member 13 and Ras-specific guanine nucleotide-releasing factor 2, and both genes have important dopamine-related functions pertaining to alcohol binge drinking. We present a hypothesis that identification of KCNK13 and RASGRF2 genes’ risk polymorphism, coupled with genetic addiction risk score (GARS)-guided precision pro-dopamine regulation, will mitigate binge alcohol drinking. Accordingly, we review published reports on the benefits of this unique approach and provide data on favorable outcomes for both binge-drinking animals and drunk drivers, including reductions in alcohol intake and prevention of relapse to drinking behavior. Since driving under the influence of alcohol often leads to incarceration rather than rehabilitation, there is converging evidence to support the utilization of GARS with or without KCNK13 and RASGRF2 risk polymorphism in the legal arena, whereby the argument that “determinism” overrides the “free will” account may be a plausible defense strategy. Obviously, this type of research is tantamount to helping resolve a major problem related to polydrug abuse.

## 1. Introduction

Excessive alcohol intake, e.g., binge drinking, is a serious public health problem in the United States and throughout the world. The fact that the rates of binge drinking are rising steadily calls for novel insights into the underlying neurobiology which may help improve prevention and therapeutic strategies [1]. Like other substance use disorders (SUDs), alcohol use disorder (AUD) is characterized by reduced dopamine signaling in the brain reward circuits, supporting its classification among other reward deficiency syndromes (RDSs) [2,3,4]. 

In the laboratory setting, the darkroom test, developed in the early 1970s, is a well-established technique to assess abnormalities of dopamine homeostasis and the efficacy of therapeutic compounds aimed at homeostatic restoration [5,6,7,8]. This procedure involves darkness-induced alcohol intake that is not only linked to melatonin-serotonergic mechanisms [6], but also to dopaminergic regulation of the brain’s mesolimbic pathways, switching neuronal expression in response to long photoperiods modulating gene expression [8]. 

It is widely recognized that increasing dopamine availability, and thus restoring dopamine homeostasis in the mesocorticolimbic system, could attenuate the motivation to seek and consume addictive substances, including alcohol [9,10]. In this regard, Solanki et al. [3] reported that the pro-dopamine regulator complex KB220 [11], administered intraperitoneally (IP) or subcutaneously (SQ), markedly and immediately reduced binge drinking of 10% alcohol (utilizing the darkroom procedure) in both male and female rats. Oral administration of KB220 was associated with a longer time period (at least three days) for the demonstration of a reliable decrease in alcohol-induced lever-pressing by both male and female alcohol-preferring rats. The same compound also decreased [3] general risk-taking behaviors [12] associated with alcohol intake [13] and other RDSs [2], e.g., activity in the open field and time spent in the open arm of the Elevated Zero Maze. As a result, this study supports KB220’s beneficial effects in reducing alcohol binge drinking in genetically-predisposed, alcohol-preferring rats. Clinical trials likewise point to the KB220 variants’ efficacy in various types of RDSs, including AUD and other classes of addictive drugs [14,15].

It is to be noted that while this article is not an exhaustive review of the entire literature, we utilized articles listed in PUBMED to organize and refer to selected items to provide rationale for our retort. When we utilized each specific gene, such as KCNK13, we found 13 items listed, and when we utilized the term RASGRF2, we found 51 items listed. Since this is a commentary and not a systematic review, only a select number of papers were cited. 

The products of two genes, namely KCNK13 and RASGRF2, respectively, code for potassium-two pore domain channel subfamily K member 13 and Ras-specific guanine nucleotide-releasing factor two, may also play an important role in alcohol binge drinking. The following sections present convergent lines of evidence suggesting that reduced KCNK13 and RASGRF2 function, due to genetic risk polymorphisms, could lead to an attenuated release of dopamine following acute alcohol administration [16]. This blunted reward response could set an individual up for alcohol and drug abuse problems and may be helpfully adjusted for by the individually tailored KB220-derived compounds. 

### 1.1. KCNK13 and Binge Drinking

Potassium (K+) leak currents’ role in neuromuscular function has been established about half a century ago [17,18]. These leak currents control neuronal excitability by shaping the duration, frequency, and amplitude of action potentials and by stabilizing the resting membrane potential. That is why suppression of leak currents enables depolarization and can cause initiation of action potentials. K+ leak currents are regulated by many chemical messengers, including but not limited to molecular oxygen, cyclic nucleotides, noradrenaline, γ-aminobutyric acid, and serotonin [19,20,21,22]. Inhibition of resting K+ leak currents by serotonin, noradrenaline, substance P, glutamate, thyrotropin-releasing hormone, and acetylcholine increases neuronal excitability in the central nervous system. In 1996, a two-P-domain channel subunit—TOK1—was found and constituted the first example of a non-voltage-gated outward rectifier [23]. It is noteworthy that KCNK0 was cloned from the neuromuscular tissues of the adult *Drosophila melanogaster*; it was found to possess the capacity to rescue potassium-transport-defective yeast cells [24]. KCNK13, first characterized by Rajan et al. [25], is a leak potassium channel that stabilizes neurons and contributes to maintaining the resting membrane potential. Specifically, two cDNAs encoding novel K+ channels, THIK-1 and THIK-2 (tandem pore domain halothane inhibited K+ channel), were extracted from rat brain; subsequently, the genes of the human orthologs were detected in human genomic database entries. They possessed one intron each and were assigned to chromosomal regions 14q24.1-14q24.3 (human (h) THIK-1) and 2p22-2p21 (hTHIK-2). As discussed below, a recent study supports the role of KCNK13 in binge drinking [26]. Variants in this gene may be involved in abnormal alcohol responses, as the response of the VTA to alcohol is dependent on KCNK13 expression [27]. However, currently, there is a dearth of information regarding polymorphisms of the KCNK13 gene, calling for more comprehensive studies regarding innate risk alleles showing reduced mRNA expression and subsequent transcription. 

A genetic predisposition, viz., the RDS feature of heightened alcohol tolerance [28], is an important etiological factor implicated in adolescent and young adult binge drinking [29]. Brain reward processing is partially a function of dopaminergic signaling, regulated by dopamine synthesis, reuptake, and degradation [30]. The dopamine transporter (DAT1), which is involved in dopamine reuptake, displays a four-times greater reuptake activity in carriers with the 9 alleles than in carriers with the 10 alleles [31]. Individuals homozygous for the COMT-*Met* allele, associated with lower enzyme activity and greater dopamine availability, demonstrate stronger alcohol-induced intoxication than those who are homozygous for the *Val* allele. In fact, the genotype combination of COMT *Val/Val* DAT *9R* is associated with blunted ventral striatal responses. These associations suggest that reduced reward sensitivity is determined partially by the aforementioned gene polymorphisms. Moreover, particular combinations of the dopamine D2 gene and the ALDH2 gene polymorphisms seem to be protective against AUD and opioid use disorder. As a result, logistic regression analysis revealed a significant interaction between ALDH2, ADH1B, and DRD2 gene polymorphisms in these patients [32]. The authors suggested that the ADH1B*1/*1, ADH1B*1/*2, and ALDH2*1/*1 genotypes may interact and guard their carriers against opioid use disorder, and the protective effect may vary relative to DRD2 gene polymorphisms. The same group also found that similar genotype combinations exhibited some protective effects against anxiety and alcoholism [33]. Additionally, it is known that people homozygous for the ALDH2 gene appear to be “protected” against binge drinking. People with the short variant of the serotonin transporter gene, which has also been implicated in RDS [34], consume more alcohol on a single occasion and become intoxicated more frequently than young adults with the normal gene variant [35,36]. 

Systematic assessment of the newer genetic and molecular neurobiological findings relevant to the physiological and psychological determinants of high alcohol consumption (including binge drinking) in animals and humans is presently ongoing [37]. While candidate gene approaches are still commonly employed to investigate associations with psychiatric disorders, Genome-Wide Association Studies (GWAS) have emphasized the multifactorial convergence entity [38,39]. For instance, second messenger genes and associated polymorphism inquiries are poised to unravel the underpinnings of binge drinking behavior, including Ras-specific guanine nucleotide-releasing factor 2 (RASGRF2), EHD4, Snapc3, and EDH1. It is noteworthy that these cited genes modulate mesolimbic dopaminergic neurons’ cell bodies in the ventral tegmental area (VTA) that send axonal projections to the ventral striatum, including the nucleus accumbens (NAc) [40,41,42]. Additionally, G protein-coupled inwardly rectifying potassium (GIRK) channels [43] are the critical regulators of neuronal excitability and affect cocaine sensitivity. Notably, potassium (GIRK) channels can be directly activated by alcohol [44,45], and GIRK3 expression in the VTA is modulated by binge drinking. Specifically, Herman et al. [46] reported that the deletion of GIRK3 in knockout (KO) mice selectively increased alcohol binge-like drinking. Also, GIRK3 KO mice showed a blunted response of the mesolimbic D-ergic pathway to alcohol, as assessed by alcohol-induced excitation of VTA neurons and dopamine release in the NAc. Second messengers and their roles in all RDS behaviors have generated a substantive interest in genetic exploration in other neuropsychiatric syndromes, including schizophrenia [47]. 

VTA neuron excitation by alcohol is inhibited by the quaternary amine, and quinidine [48] blocks diverse ion channels, including the two-pore potassium channel KCNK13. Nimitvilai et al. [49] suggested that the primary drivers of alcohol excitation of VTA neurons might be a number of ion channels, viz., h-channels (HCN) [50,51], and G protein-coupled potassium channels (GIRK) [46]. One class of ion channel, referred to as the leak K+ channel, is constitutively open and helps maintain a negative resting membrane potential [52]. 

**Figure 1:**
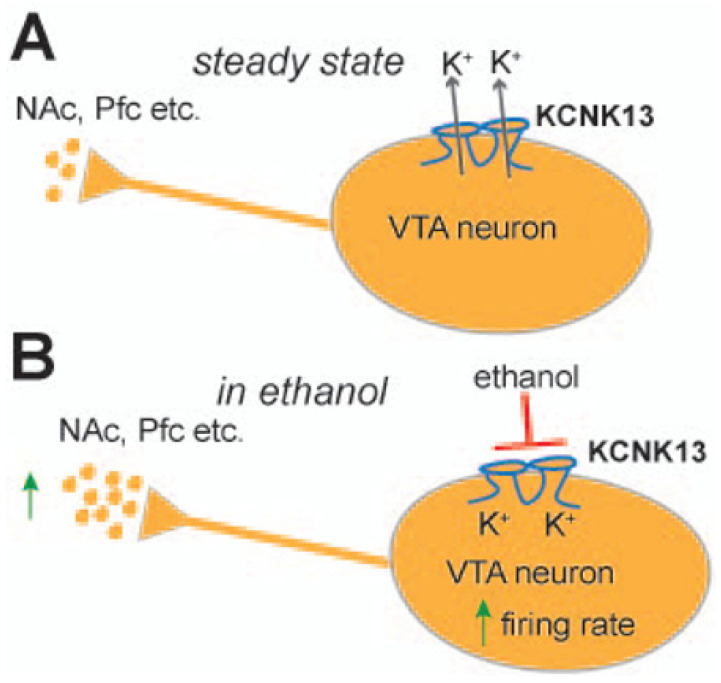
A schematic showing the steady state of VTA neurons (**A**) and how acute administration of ethanol stimulates VTA neurons by inhibiting KCNK13 (**B**); this molecule can modulate both VTA neuronal activity and binge drinking. KCNK13 is expressed in dopamine and non-dopamine neurons in the VTA. Kcnk13 gene expression is upregulated by acute alcohol consumption (Reproduced with permission from [26]).

A recent study elegantly investigated the role of the two-pore potassium channel KCNK13 and binge drinking [26]. Specifically, they found that alcohol-induced excitation of VTA neurons was selectively attenuated by shRNA targeting KCNK13. KCNK13 knockdown in the VTA also resulted in augmented alcohol intake (Figure 1). In this study, mice with decreased expression of KCNK13 in the VTA drank more alcohol than controls during the two-hour sessions, indicating the role of KCNK13 in this model of binge alcohol drinking. The effects of chronic ethanol exposure on KCNK13 have not been fully characterized in VTA, but recently it was shown that after 24 h of withdrawal after chronic ethanol exposure, there is a significant upregulation of Kcnk13 mRNA, and at 72 h of withdrawal, there is a significant downregulation of Kcnk13 mRNA. This time-dependent regulation of Kcnk13 during withdrawal after chronic ethanol exposure was reported by You et al. [27].

Since a decrease in KCNK13 channels enhances alcohol intake, a blocker of alcohol’s action on KCNK13 channels or an enhancer of KCNK13 activity may decrease alcohol-seeking behavior or even binge drinking, thus identifying a novel therapeutic target [26]. Perhaps of greater significance, there is the possibility that genetic risk polymorphisms linked to an innate deficit in KCNK13 channels, prior to any alcohol experience, may provide important information on early age vulnerability. Therefore, the identification of these polymorphisms is worthy of investigation. Furthermore, impairments caused by binge drinking or chronic alcohol intake, via these known mechanisms, support the idea of “dopamine homeostasis” [53,54,55] and its impairments in AUD.

### 1.2. Ras-Specific Guanine-Nucleotide Releasing Factor 2 (RASGRF2) and Binge Drinking 

Following a genome-wide association meta-analysis implicating the RASGRF2 gene in regulating alcohol intake in humans, Stacey et al. [56] reported that male RASGRF2^−/−^ mice exhibit attenuated alcohol consumption and preference concomitant with the perturbed mesolimbic dopamine system, which is consistent with the well-defined role of dopamine genetics in this system’s function [57,58]. 

RASGRF2 encodes a Ras-specific guanine nucleotide-releasing factor expressed across human tissues in the brain, where the expression appears to be neuron-specific [59]. It is a Ca^2+^/calmodulin-regulated protein responsible for coupling the *N*-methyl-D-aspartate and calcium-permeable α-Amino-3-hydroxy-5-methyl 4-isoxazolepropionic acid glutamate receptor–types to mitogen-activated protein kinase signaling cascades, including the extracellular signal-regulated kinase pathway [60,61]. GWAS meta-analysis of alcohol consumption helped to identify a male-specific signal in the RASGRF2 gene [62,63,64]. Schumann et al. [64] observed an association of Single Nucleotide Polymorphism (SNP) rs26907 in the RASGRF2 gene, which encodes a protein that mediates Ca^2+^-dependent activation of the ERK pathway. 

In a series of experiments in both animals and humans, Stacey et al. [56,65] discovered important phenotypic associations with the RASGRF2^−/−^ mice relative to wild-type (WT) controls. For example, alcohol-induced dopamine release in the ventral striatum was blunted in RASGRF2^−/−^ mice as was excitability in the absence of Ras-GRF2. The RASGRF2 haplotype containing rs26907 is associated with a decreased reward sensitivity and a higher number of binge drinking episodes in male adolescents. Moreover, through the ERK pathway, RASGRF2 has been shown to activate the cAMP-response element-binding (CREB) protein while also promoting long-term potentiation in the mouse hippocampus [66]. Adolescent alcohol exposure produces a persistent reduction in CREB and related signaling proteins in the amygdala and promotes high alcohol intake in rats in adulthood [67,68].

Additionally, others [41,42] have found, in a co-expression analysis, a strong correlation between α_2_ adrenoceptor RNA expression and RASGRF2 in the ventral striatum in naïve animals. Also, following acute alcohol intake, there was a reduction in β1 adrenoceptor gene expression seen in RASGRF2(^+/+^) mice; this was not observed in RASGRF2(^−/−^) mice. Conversely, alcohol resulted in a reduction in both α_2_ and β_2_ adrenoceptor gene expression in knockout mice but not in WT RASGRF2 mice. Because mesolimbic dopaminergic and extrahypothalamic noradrenergic systems are intimately linked [55], blockade of adrenergic neurotransmission via pre-synaptic α_2_ adrenoceptors’ agonists or antagonists of post-synaptic α_1_ or β adrenoceptors (e.g., clonidine, guanfacine, or prazosin) has evolved as a well-tolerated therapeutic option for AUD and other SUDs [69,70,71]. It would also be of interest to examine the potential modulation of these receptors by the KCNK13 and/or RASGRF2 products. Following identification of the known risk alleles in binge drinkers, it may be feasible to propose that the overall solution to this problem is to restore dopamine homeostasis. 

### 1.3. Precision Addiction Management for Binge Drinking Behavior 

There are about 88,000 premature fatalities in the United States annually, with a cost of about $250 billion [72], which are usually linked to excessive binge drinking and driving under the influence (DUI) of alcohol [73]. Specifically, Flowers et al. [74] reported that 84% of alcohol-impaired (AI) drivers were binge drinkers, and 88% of AI driving episodes involved binge drinkers. Interestingly, it was also found that 11.9% of binge drinkers drove within 2 h of or during their most recent binge drinking episode [75]. Park and Wu [76] found that younger age, male sex, white race, higher income, and AUD were positively associated with DUIs. Behavioral counseling or brief motivational interviews shortly after the first arrest for DUI was found to be ineffective for reducing 90-day self-reported drinking behavior and seeking treatment for drinking [77]. Nochajski & Stasiewicz [78] suggested that while there has been a decrease in the fatality rate over the past few decades, the relapse rate of DUI offenders remains quite high. Furthermore, Shaffer et al. [79] observed that repeat DUI offenders have a higher 12-month and lifetime prevalence of AUD, other SUDs, conduct disorder, post-traumatic stress disorder, generalized anxiety disorder, and bipolar disorder compared to the general population [2]. 

Though it is widely accepted that dopamine is a major neurotransmitter involved in behavioral and substance addictions, there remains controversy on how to modulate dopamine clinically, as well as how to treat and prevent various types of addictive disorders. Our overreaching assumption is that restoring dopamine homeostasis in the mesocorticolimbic system by increasing dopamine availability could attenuate the motivation to seek and consume alcohol, i.e., binging. 

**Figure 2:**
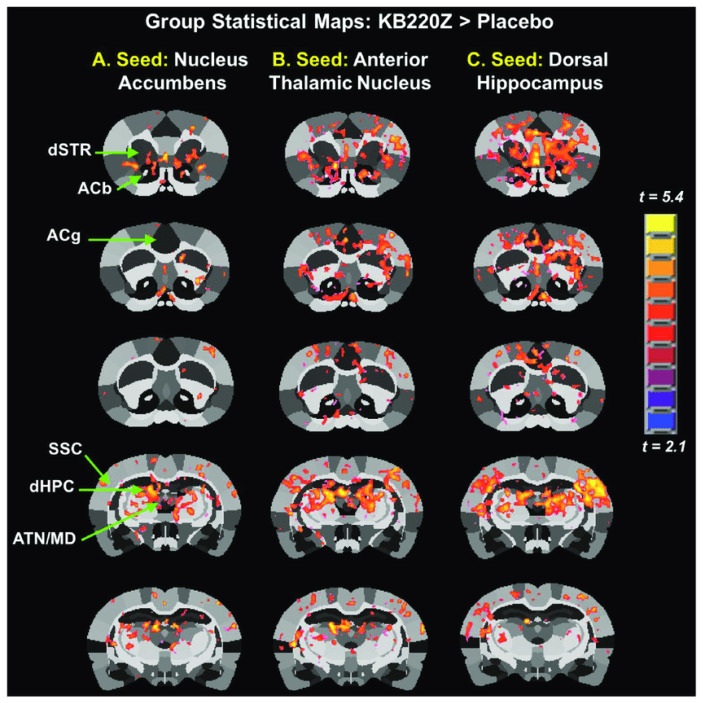
Group statistical maps comparing KB22OZ (Febo et al. [80], with permission).

It is noteworthy that alterations in synchronous neural activity between brain regions subserving reward and other cognitive functions may significantly contribute to AUD. Febo et al. [80] presented the first evidence that, in comparison to a placebo group, a pro-dopaminergic nutraceutical (KB220Z) significantly enhanced functional connectivity between reward and cognitive brain regions in the rodent model, including the NAc, hippocampus, anterior cingulate gyrus, anterior thalamic nuclei, prelimbic and infralimbic loci. Increased brain connectivity recruitment (i.e., axonal and spines’ neuroplasticity) and dopaminergic functionality were found across the brain reward circuitry. Importantly, increases in functional connectivity were specific to these regions and were not broadly distributed across the brain (Figure 2). This is important because studies on alcohol and drug effects in naïve rodents show reductions in functional connectivity after protracted withdrawal [81]. 

In earlier human experiments, Brown et al. [82] also revealed that KB220 variants specifically designed to enhance dopamine transmission, akin to the KB220z variant, significantly reduced relapse rates and enhanced recovery in DUI outpatient offenders over a 10-week period. Follow-up groups after 10 months revealed a respective 73% and 53% reduction in alcohol and cocaine intake. 

Indeed, a possible approach to attenuating binge drinking in humans may be biphasic; a short-term blockade of dopamine receptors, followed by their long-term upregulation [83]. The goal of such a strategy is to augment brain reward functional connectivity and to target reward deficiency along with the stress-like anti-reward symptomatology of addiction [84,85]. Binge drinkers’ phenotypes can be characterized using the Genetic Addiction Risk Score (GARS) that identifies reward gene risk polymorphisms across the brain reward cascade [86,87,88,89,90]. Dopamine homeostasis may thus be achieved via “Precision Addiction/Behavioral Management” (PAM/PBM), the customization of neuronutrient supplementation based on the GARS test result, along with behavioral intervention [86,87,88,89,90,91,92,93,94,95,96,97,98].

We have already performed PBM to overcome genetically-induced hypodopaminergia in a female DUI offender [96]. This case underscores the unique approach of the GARS, which is paired with a customized pro-dopamine regulator matched to polymorphic reward genes with hypodopaminergic risk potential. The proband was a female with a history of AUD and another SUD. She experienced a DUI motor vehicle accident and consequently entered voluntary treatment. Following an assessment, she was genotyped with GARS and given a neuronutrient with a KB220 base, specified by the relevant gene polymorphisms. The proband demonstrated success in recovery from all substances; she also displayed improvements in socialization, well-being, economic status, and attenuation of major depression. The urine toxicology screens were negative for at least two months from the initiation of therapy. At that time point, the patient’s parents also underwent GARS with subsequent administration of the respective KB220 variants. The proband’s father (a binge drinker) and mother (no SUD) reported improvement in various behavioral patterns, including the former’s drinking. Finally, the proband’s biological children were also GARS tested, showing a high risk for SUDs. This case series encompassing three generations is an example of the impact genetic information coupled with an appropriate DNA-guided “Pro-Dopamine Regulator” can have on recovery and enhancement of performance. Of great interest is the most recent article by Pandey’s group [99] that reported dCas9-P300 increases histone acetylation at the *Arc* SARE and normalizes deficits in *Arc* expression, leading to attenuation of adult anxiety and excessive alcohol drinking after adolescent alcohol exposure in rats. On the other hand, dCas9-KRAB increases repressive histone methylation at the *Arc* SARE, decreases *Arc* expression, provokes anxiety, and increases alcohol drinking in alcohol naïve control rats. These results show that targeted epigenomic editing using CRISPR/dCas9 can ameliorate anxiety and alcohol drinking behaviors. 

### 1.4. Determinism versus Free Will 

The discovery of the association between the *Taq A1* allele with AUD and stress [97] has been momentous in terms of trying to understand the interrelationships of DNA polymorphisms and epigenetic events. The age-old question of nature vs. nurture is beginning to be defined in terms of a balanced contribution by both. Our genomic testing center has developed the first clinically proven GARS test with PAM measuring ten reward-related genes (D1–D4, DAT1, µ opioid receptor, Serotonin transporter, GABAB3 receptor, COMT, and MAOA) and eleven SNPs. These results are coupled with a polymorphic matched pro-dopamine regulator (KB220Z PAM) to normalize “dopamine homeostasis”. 

In the fields of forensics and law, there has been intense debate regarding the implementation of DNA-directed defense, especially as it relates to defendants with antisocial personality disorder. The major argument against this defense is the paucity of evidence substantiating the role of rehabilitation in reducing recidivism in convicted criminals [98]. In contrast, the evidence for SUDs is quite robust, with many therapeutic models showing significant improvement, reasonable (spontaneous) recovery rates, and associated genetic polymorphisms linked to better clinical outcomes. One of us (RG) has successfully utilized the GARS test results as a defense argument against incarceration in the San Antonio, TX drug court for at least 16 individuals with three or more non-fatal DUIs (data in preparation for publication). To date, rather than facing prison-time of about 190 years, the defendants have been sentenced by various judges to only 170 days imprisonment and mandated probation and rehabilitation, combined with other modalities (e.g., PAM/PBM), thus sustaining the GARS genetic evidence backing the “determinism” (genetic) vs. “free will” (environment) accounts.

## 2. Conclusions

A review of the literature revealed that KCNK13 and RASGRF2 products that affect ion channels that may modulate dopaminergic function may be involved in alcohol binge drinking. In the case of a RASGRF2 haplotype containing rs26907, this SNP is associated with a decreased reward sensitivity and a higher number of binge drinking episodes. However, there is still a paucity of evidence on the KCNK13 polymorphism. On the other hand, based on data previously published, there is ample evidence that the KB220 variant significantly attenuates binge drinking in rodents and significantly reduces relapse to alcohol in human DUI offenders. In additional work, it was also shown that in a female DUI offender, subsequent testing with GARS coupled with a semi-customized precision KB220 variant therapy resulted in a significant positive clinical outcome. Furthermore, the same KB220 variant contributed to increased functional connectivity and volume across specific brain regions involved in dopaminergic function in naïve rodents. Finally, it is particularly noteworthy that ongoing research related to utilizing GARS to dissect the age-old legal question of “determinism” vs. “free-will” is now being addressed in the legal proceedings of drug court, having precedential successful conversion of incarceration to rehabilitation.
